# Biopesticides vs conventional synthetic pesticides: A multidimensional evaluation of impacts on agricultural productivity and environmental health

**DOI:** 10.1016/j.eehl.2026.100286

**Published:** 2026-09-15

**Authors:** Weitao Wang, Sarah J. Knight, Yannan Zhou, Karin Müller, Jinshu Chi, Melanie Kah

**Affiliations:** a The School of Environment, The University of Auckland, Auckland, 1010, New Zealand; b The School of Biological Sciences, The University of Auckland, Auckland, 1010, New Zealand; c Thrust of Smart Manufacturing, The Hong Kong University of Science and Technology (Guangzhou), Guangzhou, 511453, China; d New Zealand Institute for Bioeconomy Science Limited, Plant & Food Research, Auckland, 1025, New Zealand; e Earth, Ocean and Atmospheric Sciences Thrust, The Hong Kong University of Science and Technology (Guangzhou), Guangzhou, 511453, China

**Keywords:** Biopesticides, Synthetic pesticides, Quantitative comparison, Efficacy, Environmental impacts

## Abstract

Modern agriculture relies on conventional synthetic pesticides (CP) that negatively impact our environment. Derived from natural materials, biopesticides (BP) have been proposed as eco-friendly alternatives. However, there has not been a critical analysis comparing CP and BP across multiple metrics. This work systematically and quantitatively compares the agricultural productivity (efficacy, yield, and revenue) of CP and BP as well as their impacts on environmental health (non-target organisms, soil fertility, and greenhouse gas emissions) relative to untreated controls. On average, CP agronomic performance is superior (48% for BP vs 56% for CP of efficacy; 17% for BP vs 19% for CP of yield improvement), but the gap is relatively small. More than 30% of existing studies report higher efficacy and yield improvements for BP compared to CP. In addition, BP are associated with environmental benefits, including reduced non-target animal mortality (24% for BP vs 56% for CP). This work recognizes the diversity of BP and identifies key research gaps that currently hinder the broader development and adoption of alternatives to CP that could support more sustainable agricultural production.

## Highlights

• Systematic three-way comparison of biopesticides (BP) and conventional pesticides (CP).
• The difference of agricultural productivity between BP and CP is relatively small.
• BP application associates with more environmental benefits than CP.
• Identifies key evidence gaps and priorities to guide sustainable pest management.

## Graphical abstract

**Figure:**
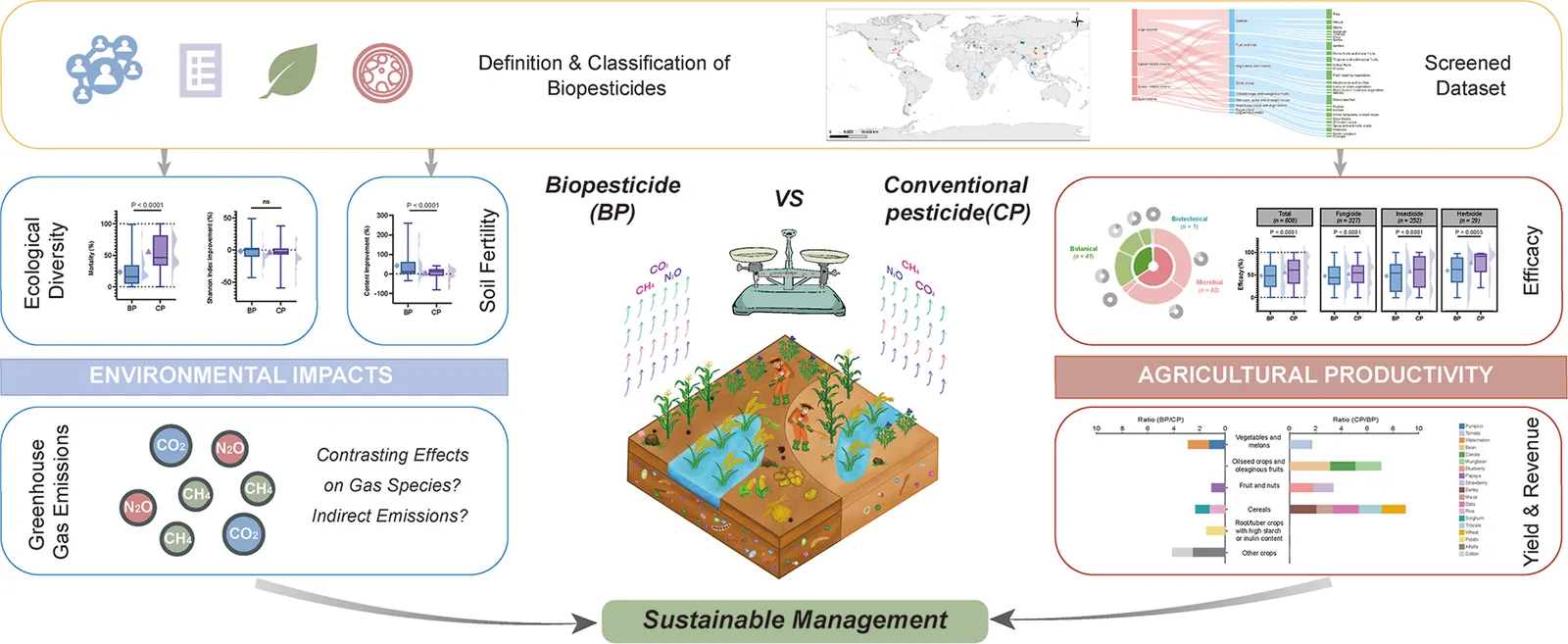

## 1 Introduction

The frequency and severity of crop pests are intensifying due to climate change, posing a growing threat to global food security [1,2]. With the global population predicted to exceed 9.8 billion by 2050, increasing agricultural productivity on limited farmland is an urgent challenge that aligns with the Sustainable Development Goals of the United Nations to eradicate hunger and poverty [3]. Addressing this challenge requires not only maximizing crop yields but also adopting sustainable pest management strategies.

As a core agricultural input, pesticides play a pivotal role in enhancing crop production under increasing biotic stresses [4]. Globally, the average pesticide application rate on cropland reached 2.49 kg/ha in 2024, nearly double that used in 1990 (Fig. 1a) [5]. Relatively high application rates are reported in countries such as Brazil and Argentina (Fig. 1b), probably due to large-scale agricultural intensification and input-dependent production systems [6,7]. Conventional pesticides (CP) consist of molecules most often produced synthetically, accounting for more than 80% of the market [8,9]. However, the intensive use of CP has led to the development of resistance in target organisms, as well as environmental contamination and impacts on non-target organisms (e.g., soil microorganisms and beneficial insects), disrupting the ecological balance [10,11].

**Fig. 1:**
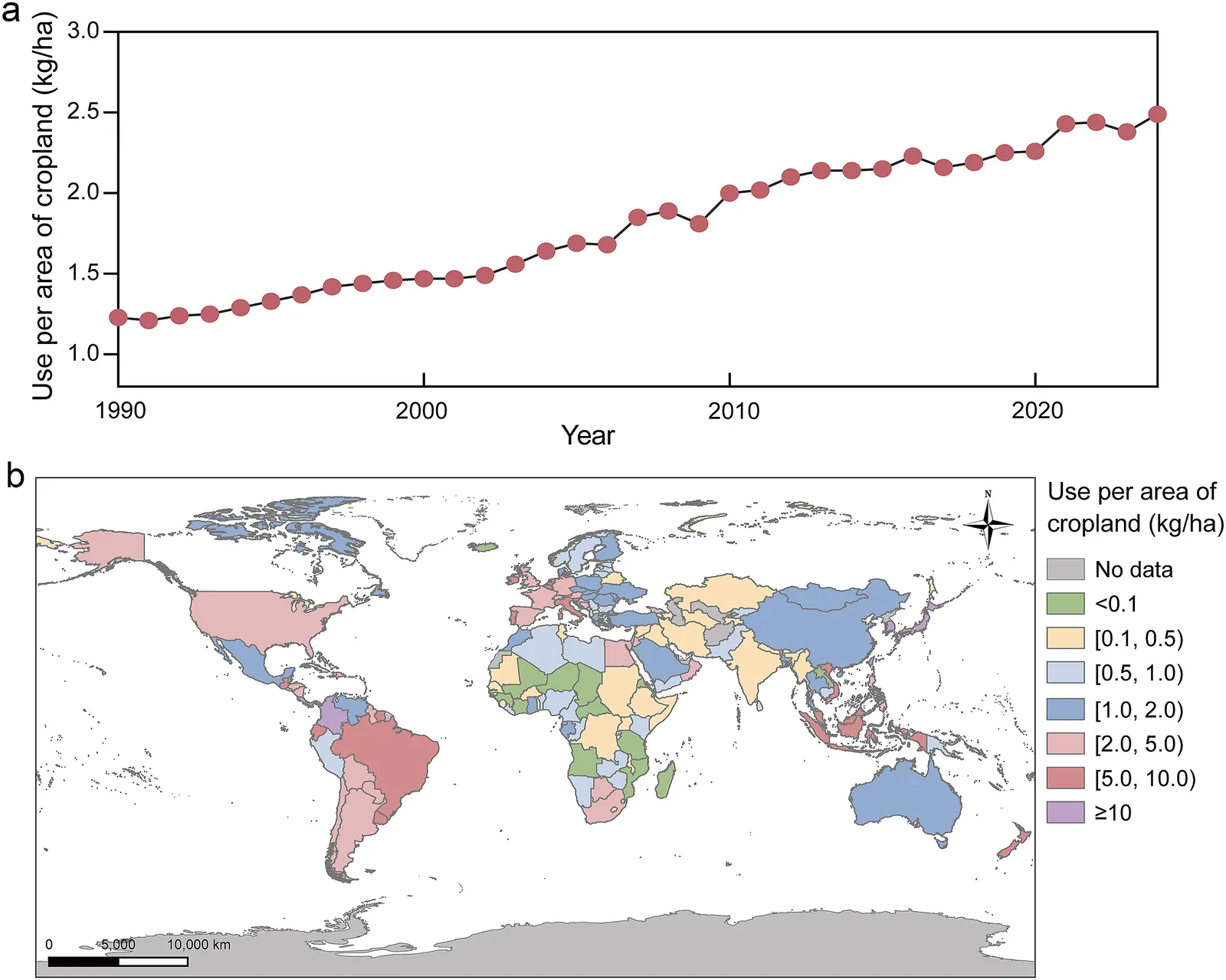
Global pesticide application in cropland. (a) Trend of global average application rate between 1990 and 2024. (b) World map of national pesticide application rates in 2024. Data were obtained from the Pesticides Use domain of Food and Agriculture Organization Corporate Statistical Database (FAOSTAT) [5].

Biopesticides (BP) are derived from natural materials (e.g., plants and microorganisms). They are generally species-selective, and their biodegradability is assumed to pose low risks to human and environmental health [[12], [13], [14]], potentially regarded as one type of alternative to CP. Nevertheless, no systematic comparative analysis has assessed the agroecological impacts of BP across multiple metrics to support (or not) this assertion in the last two decades. Our study aims to quantitatively evaluate the characteristics of BP compared to CP, focusing particularly on their effects on agricultural productivity and environmental sustainability.

## 2 Definition and classification of biopesticides

While BP are generally understood to include naturally occurring substances derived from animals, plants, or microorganisms, as well as living microbial agents that control pests, there is currently no globally accepted definition of BP [15,16]. This may be partly due to different interpretations of the term ‘bio’ by different agricultural stakeholders, with some emphasizing product-based characteristics and classifying BP as agricultural inputs, and others combining their ecological functions and regarding them as components of biological conservation. These conceptual differences are reflected in the varying classification systems adopted by different countries and organizations (Table 1). For example, natural enemies are included within the broad classification of biopesticides in China and within the Brazilian classification, whereas they are not included in the definitions adopted by Food and Agriculture Organization, the U.S., and the UK. Furthermore, the term ‘biochemical’ is subject to varying interpretations, especially regarding whether substances such as plant extracts, pheromones, and growth regulators fall within this category or warrant separate classification as botanical or semiochemical pesticides. Beyond terminology, the differences also reflect the difficulty of fitting BP into regulatory frameworks originally developed for CP, which may hinder harmonization across regions, complicate registration pathways, and slow the approval and commercialization of BP products [17].

**Table 1:** Classification of biopesticides by the Food and Agriculture Organization (FAO) and different countries.

Organization/Country	Classification						
	Microbial	Botanical/Phytochemical^a^	Semiochemical^a^	Biochemical^a^	Transgenic	Natural enemy	Other^a^
FAO [16]	✓	✓	✓				
U.S. [18]	✓			✓	✓		
UK [19]	✓	✓	✓				✓
China [20]	✓	✓		✓	✓	✓	✓
Canada [21]	✓		✓				✓
Australia [22]	✓	✓		✓	✓		
New Zealand [23]	✓	✓		✓			
Brazil [24]	✓	✓	✓	✓		✓	

a The classification of biochemical pesticides varies internationally. The U.S. defines them as naturally occurring substances that control pests through non-toxic mechanisms, including insect pheromones and plant-derived volatiles. In contrast, the UK distinguishes botanical and semiochemical products as separate categories. China, Australia, and New Zealand classify botanical pesticides separately, while grouping semiochemicals (e.g., pheromones, plant growth regulators, attractants) under biochemical pesticides. Notably, the classification shown for China reflects the broad academic definition of biopesticides. The narrow definition mainly includes microbial, botanical, and biochemical pesticides, whereas the broad definition additionally encompasses natural enemies, transgenic pest-resistant crops, and agricultural antibiotics. This broader academic definition should be distinguished from the current pesticide registration framework, under which natural enemies are exempt from registration and transgenic crops are regulated separately. Brazil categorizes only plant growth regulators as biochemical pesticides, placing pheromones and sexual attractants under semiochemicals. Canada classifies pheromones and attractants as semiochemicals, while botanical substances and plant growth supplements fall under other biopesticides.

To ensure clarity and enable comparison with CP, this paper adopts a classification system based on the source and biological functions of BP active ingredients, which are grouped into four categories: (i) microbial pesticides (living microorganisms such as bacteria, fungi, viruses, or protozoa, and their fermentation metabolites); (ii) botanical pesticides (plant extracts or secondary metabolites); (iii) biotechnical pesticides such as signaling-based agents (e.g., pheromones, kairomones), antimicrobial peptides, and RNAi-based products; and (iv) other natural-source pesticides (agents derived from other natural resources, such as mineral-based products or animal-derived compounds).

## 3 Methods

Efficacy is a fundamental criterion for evaluating crop protection products, as it refers to their ability to effectively control target pests or pathogens [25]. Many studies have measured the efficacy of BP in isolation, but direct comparisons with both the corresponding CP and untreated controls are relatively scarce. This review prioritizes efficacy as a starting point to establish a fair and structured comparison framework between BP and CP.

### 3.1 Search and screening Workflow

A multi-step literature search and screening process consisted of keyword-based retrieval, screening, and selection. First, a search was performed on 10 January 2025 in the Web of Science Core Collection, specifically the Science Citation Index Expanded (SCI-EXPANDED) edition. The Boolean query was applied to the Topic field (TS) as follows: TS = “Efficacy” AND (“Biopesticide” OR “Bioherbicide” OR “Biofungicide” OR “Bioinsecticide”). This search retrieved 691 publications published between 2004 and 2024. Reference types were limited to peer-reviewed research articles written in English. Secondly, the titles and abstracts of the selected publications were screened for relevance to biopesticide efficacy. Articles were retained only if they reported extractable numerical data and fell within the scope of the review. Specific criteria were then applied to ensure fair and robust comparisons: only studies reporting all three treatment types — a negative control (untreated), a positive control (a CP treatment) and a BP treatment — were included. There were 83 research papers identified as providing valid comparative assessments of pesticide efficacy.

Some of the screened papers also covered additional endpoints, including crop yield, natural enemies, microbial communities, and soil fertility, as well as greenhouse gas (GHG) emissions. To capture these endpoints more comprehensively, an additional search was conducted in the Web of Science Core Collection (SCI-EXPANDED) using the following TS query: TS = (“Yield” OR “Non-target Organism” OR “Beneficial Insect” OR “Natural Enemy” OR “Microbiome” OR “Microbial Diversity” OR “Fertility” OR “Soil Fertility” OR “Soil Health” OR “Soil Nutrient” OR “Greenhouse Gas” OR “CO_2_” OR “CH_4_” OR “N_2_O”) AND (“Biopesticide” OR “Biofungicide” OR “Bioinsecticide” OR “Bioherbicide”), retrieved 500 publications. Using the same screening criteria and selection procedure as described above for efficacy, this additional search identified 76 research articles with extractable numerical data that fulfilled the required three-way comparison criteria.

### 3.2 Summary of exclusion criteria

Studies were excluded if they met any of the following conditions: (1) they were not peer-reviewed research articles; (2) they were not published in English; (3) they did not report extractable numerical data; (4) they were not directly relevant to the scope of this review; or (5) they did not simultaneously include the required three-way comparison consisting of an untreated control, a CP treatment, and a BP treatment.

### 3.3 Statistical analysis

The countries and crops investigated were classified according to World Bank country classifications by income level for 2024–2025 and FAO Classification of Crops, respectively [26,27]. Each comparison was based on data generated under identical experimental conditions. BP, CP, and untreated control treatments were required to be tested within the same experiment, at the same scale and during the same period, ensuring strictly matched comparisons. Since Shapiro–Wilk’s tests indicated non-normal distributions, comparisons of studies were conducted using the Wilcoxon matched-pairs signed-rank test in GraphPad Prism (v10.5.0; GraphPad Software, Boston, MA, USA). Details on the calculation of each parameter are presented in Table S1.

## 4 Results and discussion

The distribution, experimental locations, and crop categories represented across the 118 selected studies are presented in Fig. S1. The selected papers covered multiple research themes, including efficacy (*n* = 83) and yield (*n* = 45), with comparatively fewer studies considering non-target animals (*n* = 16), non-target microorganisms (*n* = 12), greenhouse gas (GHG) emissions (*n* = 5), and soil fertility (*n* = 3) (Fig. S1a). Most work was conducted in high-income and upper-middle-income countries (Fig. S1b), primarily focusing on cereals, fruit and nuts, and vegetables and melons (Fig. S1c). The following discussion is structured around two domains: agricultural productivity (including efficacy, crop yield improvement and revenue) as well as environmental sustainability (including impacts on natural enemies, soil microbial diversity, soil fertility, and GHG emissions). To provide a better understanding of the differences between BP and CP, the results of agricultural productivity are presented first at an overall level and then broken down into the main categories of pesticides (fungicides, insecticides and herbicides).

### 4.1 Efficacy

#### 4.1.1 Different types of alternatives to conventional pesticides

Microbial pesticides account for the majority of products reviewed across 608 studies (i.e. individual comparisons between CP and BP; a paper typically presents several studies) in 83 papers meeting the selection criteria (Fig. 2a), with *Bacillus* and *Trichoderma* dominating. Among the botanical pesticides, neem and its active ingredients were the most widely studied. The only biotechnical pesticide identified was a spider venom peptide. Regarding commercial status, 70% of the BP products were commercially produced. In terms of target pests, biofungicides (46.7%) and bioinsecticides (44.3%) were the common categories tested, whereas bioherbicides constituted a minor share of 9.0% (Fig. 2a). This disparity is particularly notable given that the global application of synthetic herbicides far exceeds that of insecticides or fungicides [28]. The scarcity of bioherbicide products is further evidenced by the small number of these substances listed in the EU pesticide database [29]. Compared with pathogens and insects, weeds pose greater challenges for biopesticide development due to their high physiological diversity, complex resistance mechanisms — including target-site mutations (e.g., in acetyl-CoA carboxylase or acetolactate synthase enzymes) and non-target-site metabolic resistance — and the difficulty of achieving crop-weed selectivity [30,31]. In addition, some bioherbicides, especially microbial bioherbicides, often target only a narrow range of weed species, thereby limiting their market appeal compared to broad-spectrum chemical alternatives [32]. These biological constraints, coupled with limited market incentives and high development costs, are probably the main hurdles limiting the current development of bioherbicides [33].

**Fig. 2:**
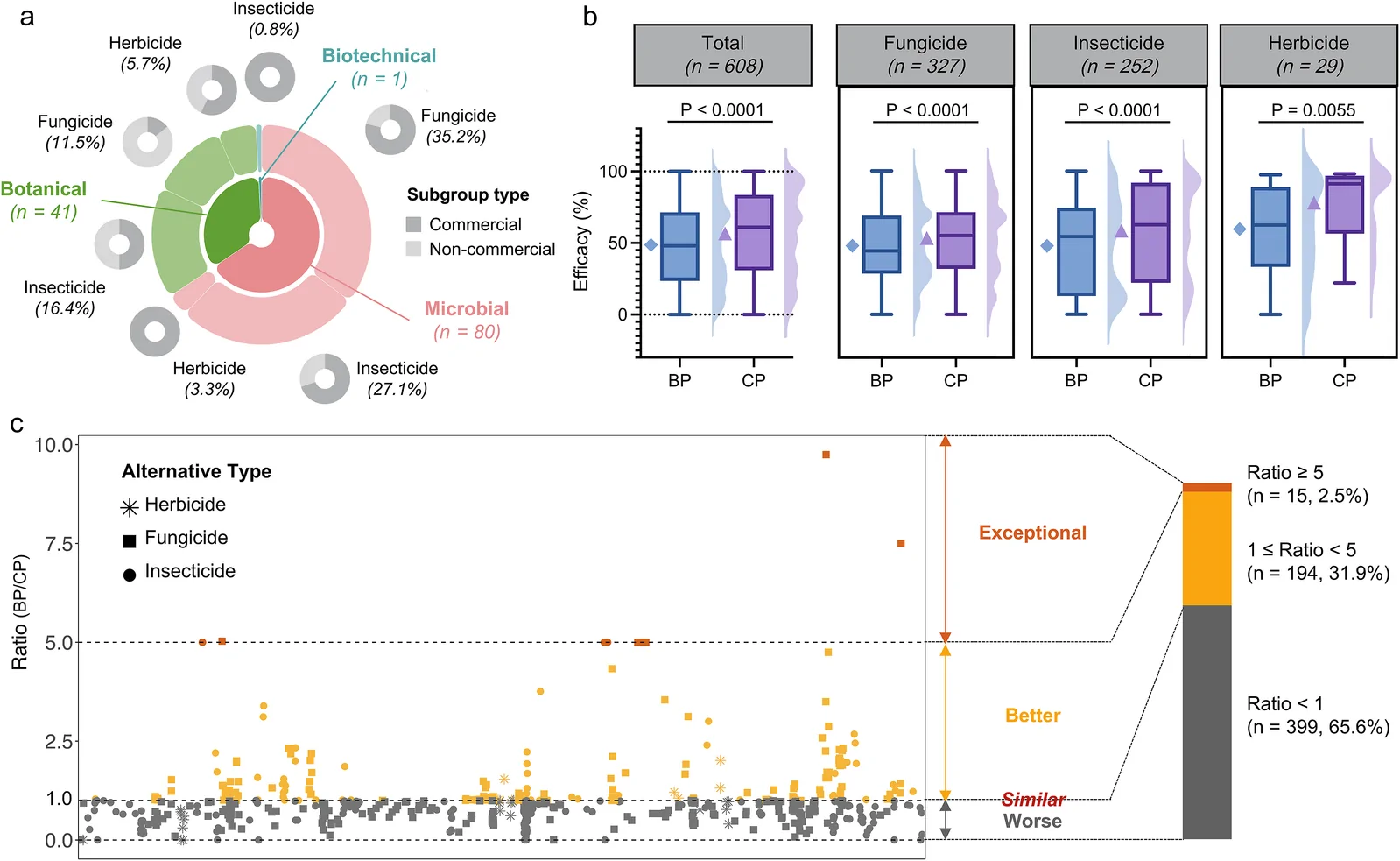
Comparative efficacy analysis based on 608 studies from 83 published papers. (a) Classification and commercial status of investigated biopesticides (BP). (b) Comparison of efficacy between BP and conventional pesticides (CP). Diamonds and triangles represent the mean efficacy of BP and CP, respectively. Each box plot presents the median (horizontal line) and interquartile range (box). (c) Distribution of efficacy ratios (BP efficacy/CP efficacy). A ratio < 1 indicates a lower efficacy of the BP.

Efficacy comparisons suggest a significantly lower absolute efficacy of BP (48.4% ± 28.9%) compared with CP (56.5% ± 29.7%) (*P* < 0.0001) (Fig. 2b, Table S2). Specifically, the median and mean of conventional fungicide efficacy (55.3% and 53.0%, respectively) were significantly higher than those of biofungicides (44.3% and 47.7%, respectively) (*P* < 0.0001). The greater variability and overall lower efficacy of biofungicides were primarily driven by a subset of products with markedly lower efficacy (Fig. 2b, Table S2). This suggests that while some biofungicide products clearly underperform, others approach the efficacy levels of conventional fungicides. The efficacy gap between bioinsecticides and conventional insecticides was more pronounced than that observed for fungicide treatments. The limited data on bioherbicides suggest that their efficacy was significantly lower than that of conventional herbicides (59.4% for BP vs 77.7% for CP; *P* = 0.0055) (Fig. 2b, Table S2).

When directly compared to their conventional counterpart within a given study, efficacy ratios also indicate the lower efficacy of BP (Fig. 2c). For more than 20% of the studies, BP were obtained through grinding plant materials for aqueous preparations or using Soxhlet extraction to obtain essential oils [[34], [35], [36], [37]]. Although these simple approaches increase the accessibility of BP, they might lead to relatively low concentrations and stability of bioactive compounds, which may explain their relatively low efficacy. Comparisons between lab-produced BP and commercial CP should be interpreted with care as the latter benefit from optimized formulations containing multiple additives that enhance their properties (e.g., rainfastness and stability). The lack of optimized formulation may be compensated by excessive application rates, which may not be viable under realistic conditions and lead to misleading conclusions during comparisons with CP, not only in terms of efficacy, but also in terms of costs and potential undesired impacts.

Overall, 34.4% of the comparisons reported similar or greater performance of BP (mainly microbial pesticides) compared with CP. For instance, the commercial biocontrol product Vintec (*Trichoderma atroviride*) achieved exceptional control (100% efficacy) of *Neofusicoccum parvum*—a major pathogen associated with grapevine trunk diseases—whereas the efficacy of the conventional fungicide myclobutanil was only 10.3% in a vineyard [38]. Similarly, the commercial product Tricotrap® (*Trichoderma harzianum*) exhibited a 7.5-fold higher efficacy than thiophanate-methyl in the control of wood diseases caused by *Botryosphaeriaceae* in a commercial pear orchard [39]. In comparison to the conventional fungicide mancozeb, which was banned by the European Commission in 2021 [40], several microbial fungicides—such as *B. amyloliquefaciens* MBI 600, *B. subtilis* QST713 and *E. cloacae* B-21050—showed greater control of the common scab in potato and crown rot in Gerbera [41,42]. The greater performance of microbial biofungicides can be attributed to their ability to multiply in the target environment after application, thereby increasing persistence and long-term activity compared to botanical pesticides [43,44]. Moreover, microbial pesticides can achieve relatively high efficacy by utilizing multiple modes of action associated with their ecological niches and biological characteristics [45]. Unlike CP, which typically targets a single biochemical pathway, a microbial agent can simultaneously employ direct modes of action—such as antibiosis, parasitism, and signaling disruption—and indirect mechanisms, including niche competition and the induction of systemic plant resistance [17]. For instance, *Bacillus* species can suppress disease by disrupting pathogen membranes through lipopeptide production, while also activating the host’s immune system by inducing systemic resistance [17].

#### 4.1.2 From the laboratory to the field

Efficacy comparisons between BP and CP across multiple scales are crucial to critically assess their performance gap. At the laboratory scale, BP showed higher, though not statistically significant, mean efficacy (69.8%) compared with CP (67.2%) based on 31 comparisons presented in 12 papers (*P* = 0.5275) (Fig. 3a, Table S3). In contrast, significantly higher efficacy was reported for CP at semi-field (e.g., greenhouse, polytunnel, and screenhouse) and field scales (Fig. 3b and c). This discrepancy could be attributed to laboratory conditions that oversimplify ecological interactions and increase physiological stress on target pests [46], thereby potentially overestimating BP efficacy. For instance, *Isaria fumosorosea* Wize, the BP used against rugose spiraling whitefly, showed 1.6-fold higher efficacy than the CP buprofezin under laboratory conditions [47]. However, this advantage was not maintained in field trials. The authors suggested that the discrepancy was due to the pests mainly residing on the lower leaf surface, leading to less effective spray coverage in the field compared to the laboratory. The waxy cuticular lipids on the host plant may also limit blastospore adherence and germination, leading to the lower efficacy of the BP compared with the laboratory scale [47]. In addition, environmental variability in field trials—such as temperature and UV radiation—can strongly affect BP performance. For instance, *S. carpocapsae*, a commonly used bioinsecticide for controlling spotted wing *Drosophila* (*Drosophila suzukii*), achieved 85.8% efficacy under constant temperature (25°C), but efficacy declined to 28.3% under fluctuating temperatures (11–22°C) [48]. In comparison, the high efficacy of CP deltamethrin and spinosyns against *Drosophila* was not affected by temperature (14, 22, and 30°C) [49]. Similarly, the efficacy of azadirachtin-based BP can be limited by their poor pH stability and high sensitivity to photodegradation because of the presence of light-absorbing moieties [50]. Some interactions in the crop production system, such as competition between microbial pesticides and resident microorganisms and crops within limited resource availability, may also reduce the efficacy of BP under field conditions [[51], [52], [53]]. Therefore, some advantages of BP (e.g., inherent specificity and rapid degradation) act as a double-edged sword, limiting environmental impacts but also limiting their efficacy [54]. This also underscores the potential for improving BP performance through formulation optimization, particularly when the specific examples involved unformulated BP.

**Fig. 3:**
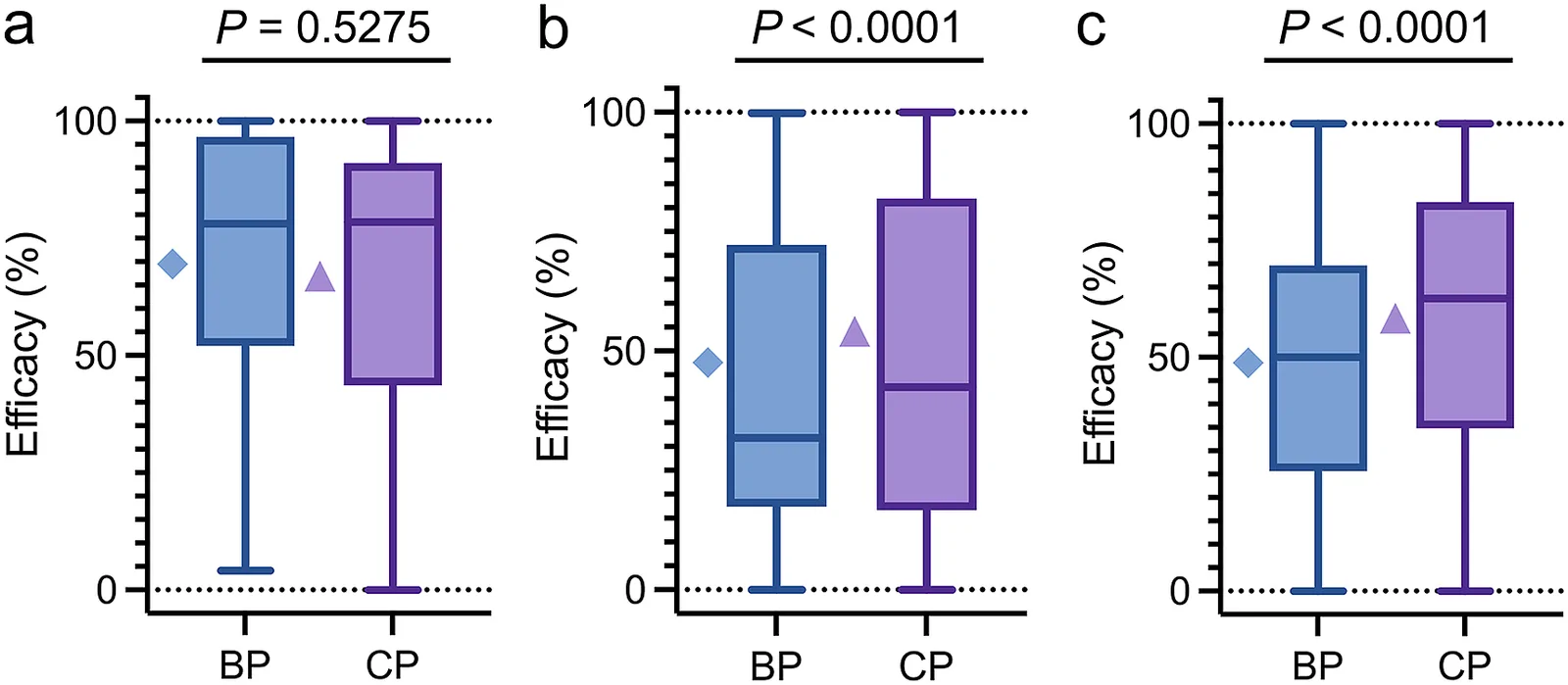
Comparative efficacy analysis between biopesticides (BP) and conventional pesticides (CP) at different scales. (a) Laboratory scale (*n* = 31). (b) Semi-field scale including greenhouse, polytunnel, and screenhouse (*n* = 181). (c) Field scale representing fully open environmental conditions (*n* = 396). Diamonds and triangles represent the mean efficacy of BP and CP, respectively. Each box plot presents the median (horizontal line) and interquartile range (box).

### 4.2 Yield and revenue

Crop yield per unit area is a critical socioeconomic endpoint when evaluating different management practices on limited land resources [55]. Based on 353 comparisons extracted from 45 papers, microbial pesticides constitute the dominant category of the investigated BP, most of which were applied as alternatives to conventional fungicides (Fig. 4a). Regarding yield improvement, although the average improvement level for CP was significantly higher than that for BP (*P* < 0.0001) (Fig. 4b, Table S4), the absolute difference between them was relatively small (16.5% for BP vs 19.2% for CP). Median yield improvements for the bioinsecticide and conventional insecticide treatments were very similar (5.8% and 5.7%, respectively). Although conventional insecticides (17.5%) supported greater average improvement than bioinsecticides (14.8%), the difference was not statistically significant (*P* = 0.3067) (Fig. 4b, Table S4). Similarly, conventional herbicides showed higher mean yield improvement than bioherbicides with no significant differences (39.3% for BP vs 44.1% for CP) (*P* = 0.8438) (Fig. 4b, Table S4).

**Fig. 4:**
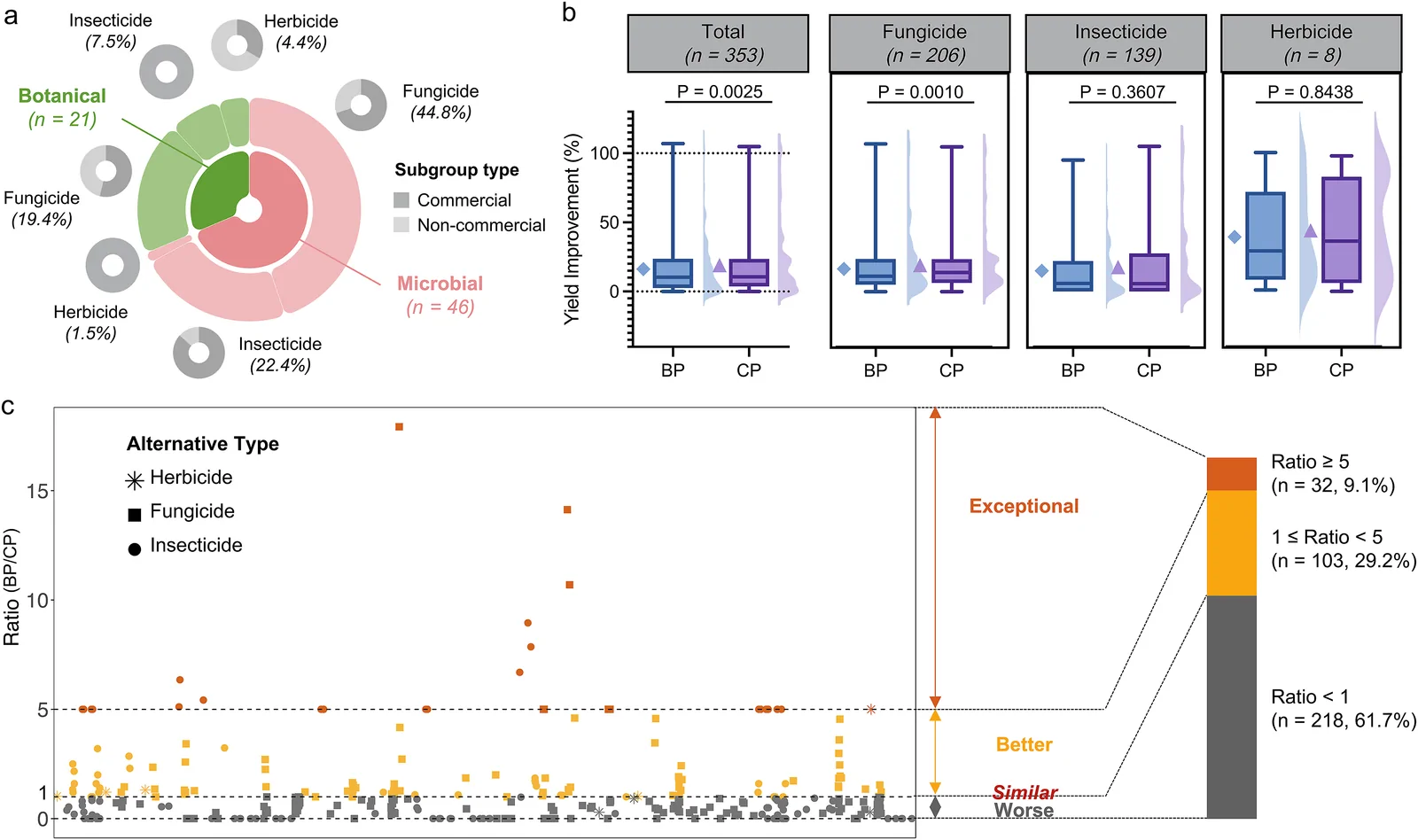
Comparative yield improvement analysis based on 353 comparisons from 45 published papers. (a) Classification and commercial status of investigated biopesticides (BP). (b) Comparison of yield improvement between BP and conventional pesticides (CP). Diamonds and triangles represent the mean of BP and CP, respectively. Each box plot presents the median (horizontal line) and interquartile range (box). (c) Distribution of yield improvement ratios (yield improvement of BP/yield improvement of CP). A ratio < 1 indicates a lower yield improvement of the BP.

Based on yield ratios, 61.7% of the studies reported better performance for CP compared to BP (Fig. 4c). For instance, under the stress of *Rhizoctonia solani*, a major fungal pathogen causing sheath blight, the combination of the CP trifloxystrobin and tebuconazole increased rice yield by 23.4%, compared with a BP mixture of cinnamon and clove oil (11.3%) [37]. Similarly, the broad-spectrum conventional insecticide imidacloprid resulted in a 73.2% yield increase compared with the BP Spinosad (36.7%) in a canola field infested with crucifer flea beetles [56]. However, most of these papers cite similar cases to validate the superior yield of pesticides without investigating the underlying mechanisms responsible for such differences compared to BP. About 38% of the studies reported greater yield improvements when using BP compared with CP, with several biofungicides and bioinsecticides showing exceptional performance (Fig. 4c). For example, the BP *B. subtilis* QST713 and mustard meal, both effective against common scab, improved potato yields by 32.5% and 24.6%, respectively, compared to the CP MnSO_4_ (0%) and mancozeb (2.3%) [42]. In cotton production, *Beauveria bassiana* achieved superior control of spider mites, leading to a seven-fold increase in yield relative to the CP lambda-cyhalothrin [57]. Another example is that the application of *M. anisopliae* ICIPE 62 resulted in higher yields than the CP duduthrin, which showed no yield improvement across several growing seasons in aphid-infested cowpea fields [58]. The poor performance of CP could be caused by resistance in target organisms due to repeated long-term use. BP can help break this resistance cycle because they control diseases via diverse modes of action, potentially further increasing crop yields [[58], [59], [60]]. Moreover, BP may promote crop growth and protection through biofilm formation and systemic resistance against target organisms [61,62], unlike CP, which can induce oxidative stress and metabolic disruption in crops [63].

To evaluate the potential economic returns of BP and CP, revenue increases were compared in a crop-specific context based on their average yield improvements together with crop-specific base yield and unit price [26,64]. Only studies in which both BP and CP were commercial products were included to ensure objective revenue comparisons. Overall, while yield differentials primarily dictated the direction of revenue response, the magnitude was governed by crop-specific base yields and market pricing. As shown in Fig. 5, CP produced greater yield improvements for most crops, particularly cereals and oilseed crops and oleaginous fruits, while BP outperformed CP in root/tuber crops and other crops. When crop-specific base yield and market price were considered, the economic effects of these yield differences became more pronounced (Table S5). BP generated greater estimated revenues for crops such as papaya, alfalfa, and potato, while CP remained more advantageous for crops such as strawberry and blueberry.

**Fig. 5:**
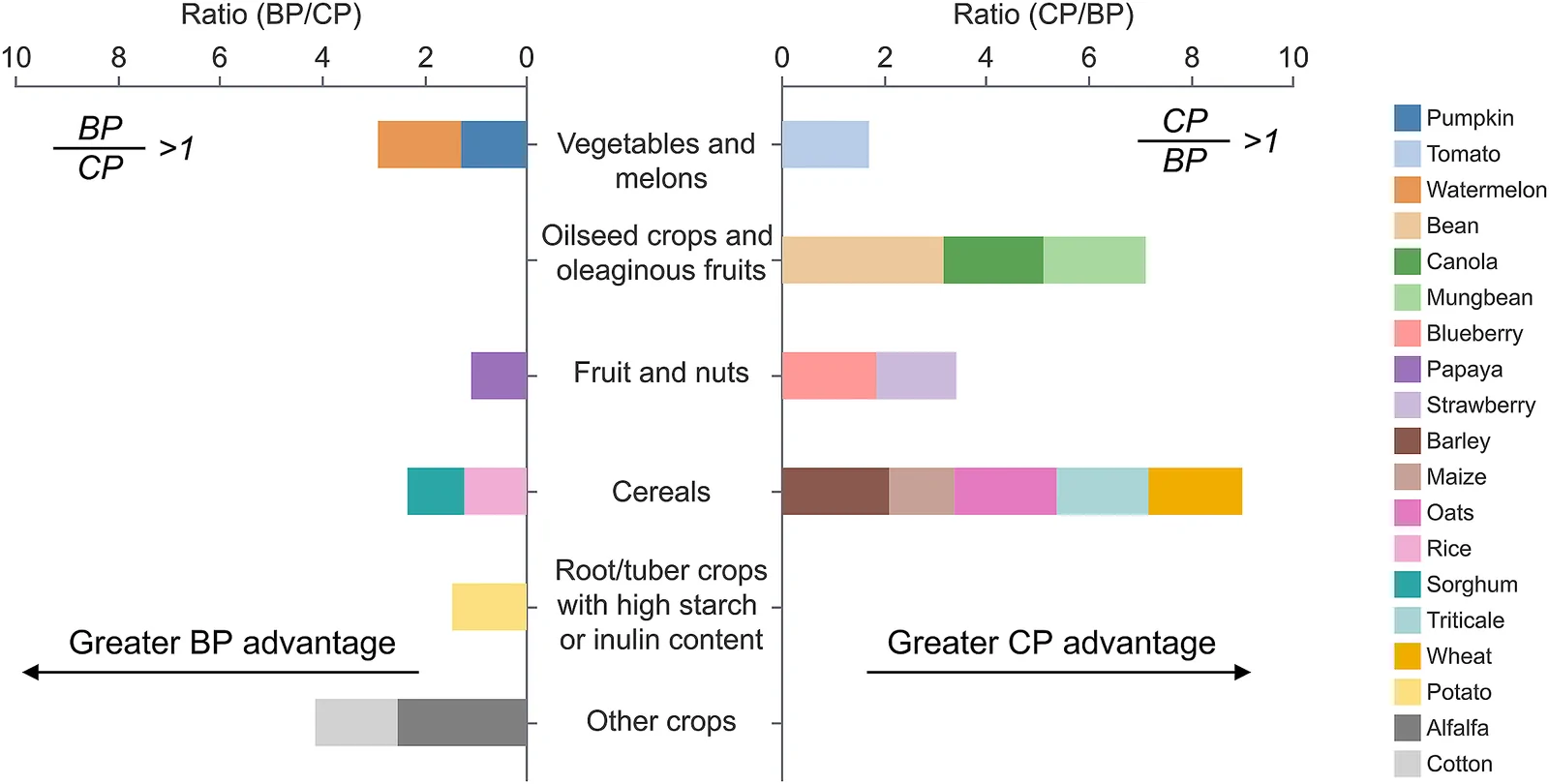
Average yield improvement ratio supported by the application of biopesticides (BP) or conventional pesticides (CP) for different crops. The left panel shows crops for which BP are superior, while the right panel shows those for which CP are superior.

Most of the reviewed papers did not report the costs of BP and CP products, which limited the ability to perform a comprehensive cost–benefit analysis. The few studies considering both costs and revenues reported that CP was more profitable since BP generally had higher costs due to more frequent applications [[65], [66], [67], [68]]. However, this pattern was not always consistent. For instance, in a cabbage case study, botanical BP derived from Siam weed and tobacco generated higher benefits than the synthetic insecticide Attack® during the major rainy season. This advantage changed in the minor rainy season, where Attack® only slightly outperformed those botanical treatments [69]. These findings indicate that studies integrating both costs and benefits are still critically needed. Nevertheless, such quantitative analyses mainly capture short-term revenue. The sustainable benefits (e.g., less disruption to the environment) potentially supported by BP should be further investigated to better assess their economic viability in both short and long terms [14].

### 4.3 Environmental impacts

Besides controlling target pests and diseases, CP and BP can lead to non-target impacts in the receiving environment, such as toxic effects on non-target organisms and deterioration of soil health [10,70]. A broad understanding of the differences in non-target effects between BP and CP can inform the development of more sustainable agricultural practices.

#### 4.3.1 Non-target animals

Non-target animals, including pest natural enemies and beneficial insects, provide essential pest management services and support agricultural productivity [71]. Assessing the compatibility of BP and CP with these beneficial animals is crucial for assessing ecological impacts [72]. Data from 160 comparisons across 16 papers indicated that BP generally caused significantly less mortality to these beneficial organisms, as evidenced by a significantly lower average mortality (23.5% vs 56.2%, P < 0.0001) compared to CP (Fig. 6a, Table S6). For instance, the conventional insecticide endosulfan resulted in larger reductions in non-target and potentially beneficial spiders, lacewings and green ants (85.8%, 70.8% and 69.6%, respectively) than neem oil (43.2%, 31.5% and 25.3%, respectively) in a mango field infested by mango hoppers (*Idioscopus clypealis* L) [73]. Another study reported that the mortality of the beneficial organism, *Cleruchoides noackae* adults, exposed to bifenthrin (conventional insecticide, 72.6%) was much higher than the mortality induced by *Metarhizium anisopliae* (bioinsecticide, 4.6%) in *Eucalyptus* production [74]. Nevertheless, BP cannot always be assumed to be less disruptive to natural enemies. For example, Spinosad (BP) exposure led to a higher reduction in parasitoid *T. brassicae* emergence (94.8%) compared with phosalone (CP, 84.1%), which is a crucial biological agent of *Lepidoptera* pests worldwide [75]. Besides, imidacloprid (CP) and Azamax™ EC (an azadirachtin-based BP) resulted in high adult mortality of the ectoparasitoid *T*. *radiata* (84.7% and 73.6%, respectively) [76]. Overall, while BP generally cause lower mortality in beneficial organisms than CP, their impacts vary across agents and species.

**Fig. 6:**
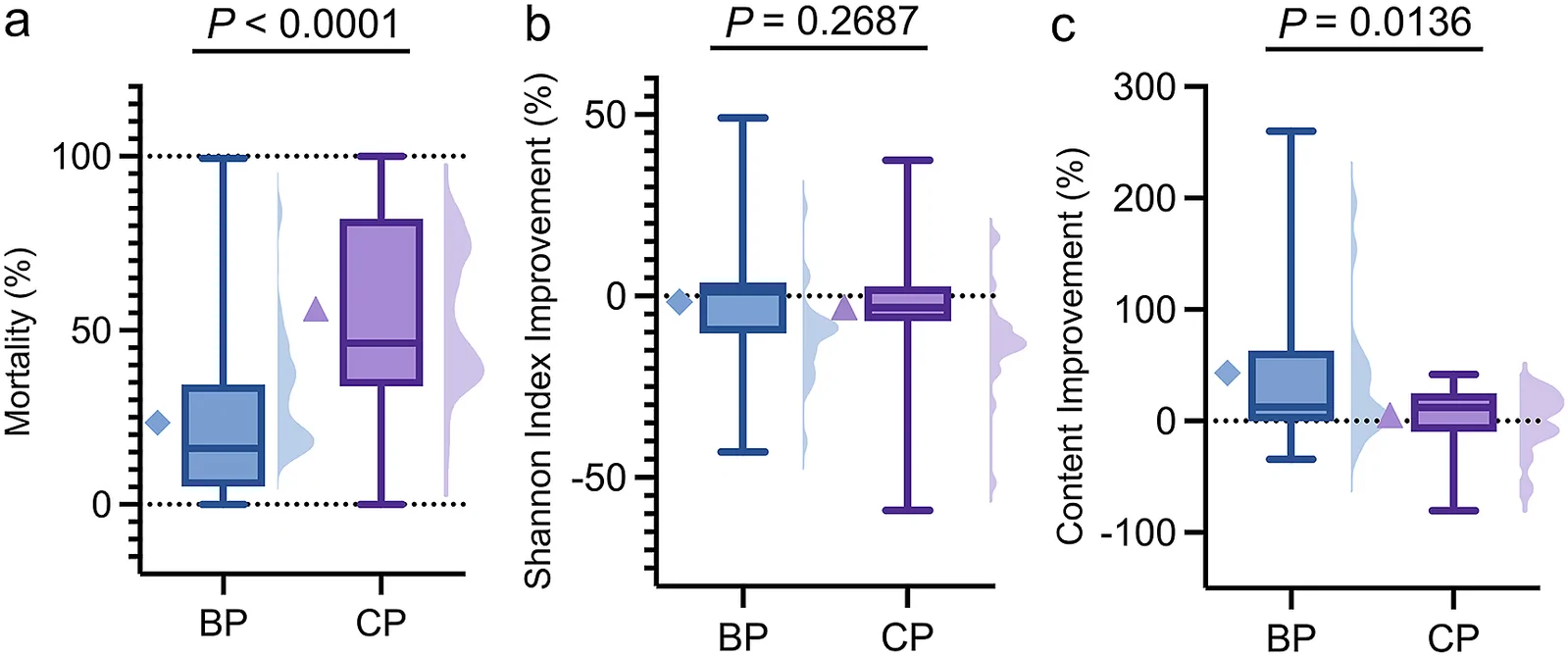
Non-target impacts of biopesticides (BP) and conventional pesticides (CP) on mortality of (a) non-target animals (*n* = 160), (b) microbial diversity (*n* = 27) and (c) soil fertility (*n* = 20). Diamonds and triangles represent the mean values of different parameters of biopesticides and conventional pesticides, respectively. Each box plot presents the median (horizontal line) and interquartile range (box).

#### 4.3.2 Non-target microorganisms

Soil microorganisms play a central role in mediating multiple ecosystem processes (e.g., nutrient cycling and soil structure formation) and contribute to soil health [77]. The effects of BP and CP on non-target microorganisms are discussed here at two levels: taxon-specific abundance and alpha diversity of microbial communities.

At the individual taxon level, most available evidence focused on microbial pesticides. For example, *Pseudomonas aeruginosa* and *Lasiodiplodia pseudotheobromae* increased the absolute abundance of heterotrophic and nitrogen-cycling bacteria by over 20%, whereas the conventional counterpart glyphosate caused reductions exceeding 10% [78]. Similarly, the BP *Bacillus subtilis* increased the proportion of native phylloplane bacteria and fungi antagonistic to *F. verticillioides* by more than 30% relative to the untreated control, whereas the CP treatments (cyproconazole and azoxystrobin) reduced these proportions by more than 32% [79]. However, it should be noted that data regarding the impact of botanical pesticides and other BP on specific microbial guilds remain scarce.

For the effects on microbial diversity, a total of 27 studies from 10 papers showed that the differences of Shannon diversity of microbial communities were not significant (−1.3% for BP and −2.8% for CP; *P* = 0.2687) (Fig. 6b, Table S6). More specifically, the bacterial diversity presented smaller changes (1.1% for BP and −2.4% for CP; *P* = 0.1964) (Fig. S3a), whereas the fungal diversity exhibited declines, averaging reductions of 6.4% and 3.6% under BP and CP treatments, respectively (*P* = 0.9102) (Fig. S3b). Compared with the clear differences observed in specific microbial groups, the relatively small changes of Shannon diversity may reflect the resilience of microbial communities under pesticide stress [80,81]. Nevertheless, alpha-diversity responses may not adequately capture the concomitant functional alteration due to different modes of action. For example, although a conventional fungicide (fosetyl-Al and propamocarb-hydrochloride) and a microbial pesticide (*Clonostachys rosea*) had minimal effects on soil microbial alpha diversity, both markedly altered community composition, reduced network complexity, and affected soil functions through different modes of action [82]. Specifically, the BP influenced keystone taxa structuring the microbial network, whereas the CP altered biotic interactions, promoting taxa that are less efficient in organic compound degradation. These results highlight a key knowledge gap: diversity metrics provide useful first-order indicators, but they fail to capture functional reorganization and ecological consequences. A more function-oriented framework is needed to differentiate how BP and CP alter microbial processes and ecosystem stability.

#### 4.3.3 Soil fertility

Soil fertility is essential for crop growth and overall productivity [51]. Data from 20 studies across 3 papers showed that BP significantly increased soil nutrient contents to a greater extent than CP (43.4% vs 5.4%; *P* = 0.0136) (Fig. 6c, Table S6). For example, the treatment with *S. pseudogriseolus* sp. S-9 and *R. irregularis* resulted in a 100% increase in total phosphorus of cultivated *Cajanus cajan* soil, compared to a 25% increase with the conventional fungicide Bavistin in a pigeon pea farm under *Fusarium* wilt stress between 2018 and 2019 [83]. In several laboratory studies, soil under BP treatments, such as 2-(hydroxymethyl) phenol, *Pseudomonas aeruginosa C1501*, and *Lasiodiplodia pseudotheobromae C1136*, presented higher organic carbon levels than soil treated with the synthetic herbicide glyphosate [78,84]. These studies suggest that BP may promote soil nutrient availability by producing bioactive compounds that stimulate microbial processes such as nitrogen fixation and phosphate solubilization [85,86]. Nevertheless, recognizing the complexity of soil-pesticide interactions and the limited availability of field-scale data, these preliminary findings should be interpreted with caution. Further research is essential to confirm whether the benefits associated with BP application are reliable under realistic conditions.

#### 4.3.4 Greenhouse gas emissions

Agriculture contributes approximately 12% of global anthropogenic GHG emissions—including carbon dioxide (CO_2_), methane (CH_4_), and nitrous oxide (N_2_O)—which are key drivers of climate change and thus pose significant threats to global food security [2,87]. As BP and CP can alter microbial activity and nutrient cycling in soils, their applications can further affect soil and ecosystem GHG exchanges with the atmosphere [88,89]. Several studies showed contrasting effects between BP and CP on soil GHG emissions, depending on the gas species, study scales, and experimental periods. For example, in a 7-day laboratory incubation with urea-amended aerobic soil [90], treatments with both *G. sepium* leaf extracts (BP) and lambda-cyhalothrin (CP) increased CH_4_ emissions by 50%, reduced CO_2_ emissions by 12% relative to the control, but had no effect on N_2_O emissions. However, in another two-month study, greenhouse experiments on bean-cultivated soil revealed an increase in cumulative CO_2_ emissions following BP application, though no significant differences in cumulative N_2_O emissions between treatments were observed compared with the conventional counterpart [91]. Similarly, compared with untreated soil, 2-(hydroxymethyl) phenol (bioherbicide) significantly increased CO_2_ flux by 40%, while significant reductions (−37%) were observed in the soil treated with glyphosate (conventional herbicide) after a 70-day laboratory incubation [84]. The observed increase in GHG emissions by BP was attributed to enhanced microbial enzyme production followed by accelerated nutrient cycling [84]. These studies on GHG emissions are largely limited to observations within well-controlled conditions and short experimental periods. Given the potential of BP to alter microbial-mediated biogeochemical cycles, long-term and large-scale monitoring (e.g., several growing seasons at an ecosystem scale) of their effects on microbial communities and GHG dynamics would help inform more sustainable soil management [92].

Considering the broader life cycle of pesticides, the production of CP involves energy-intensive processes, often requiring high temperatures and pressures, as well as substantial fossil fuel inputs [93]. Emissions from these industrial processes also constitute a major component of the global GHG inventory. By contrast, BP manufacturing typically uses agricultural raw materials or waste products and operates under milder conditions, potentially resulting in lower energy requirements and the associated GHG emissions [[94], [95], [96], [97]]. For example, the production of the amphidinol-biobased fungicide showed a reduction of more than 80% in global warming potential as well as less human toxicity compared with some commercial fungicides (Priaxor®, Delan® Pro) [98]. However, a comprehensive assessment of GHG emissions should also consider indirect and system-level emissions associated with pesticide-enabled management. For example, compared with water treatment, botanical and microbial pesticides (e.g., essential oils of plants and nitrogen-fixing bacteria) could potentially reduce more than 20% energy consumption and CO_2_ emissions during shallow soil tillage [99]. It is also well recognized that the high efficacy of systemic herbicides (e.g., glyphosate) has facilitated the global adoption of no-till or reduced-tillage practices, which significantly decrease fossil fuel consumption by farm machinery and topsoil loss [100]. Comparing CP and BP will require a more holistic consideration of agronomic practices and their impacts at the ecosystem level and beyond.

## 5 Conclusions and recommendations for future work

This study showed that CP generally achieved higher efficacy and yield improvement than BP, although the differences between them were relatively small. Notably, BP were often associated with fewer environmental impacts than CP. However, it should be recognized that BP cannot be regarded as risk-free, with existing examples of greater negative effects on non-target organisms for BP compared to CP. Given the variation in environmental impact quotient (EIQ) values among CP [101], more studies comparing BP with multiple relevant CP and an untreated control across efficacy, economic and environmental impacts are required to support the robust evaluation of their performance and associated risks and benefits. Furthermore, a greater understanding of the modes of action of BP will facilitate the identification of potential non-target impacts.

Research should continue to explore existing and novel bioactive ingredients (including substances from plants, microorganisms and other natural sources and double-stranded RNA) and improve their performance through well-designed formulations (e.g., synergistic formulations) to enable more diverse, selective and efficient applications. Inconsistency between results generated in the laboratory and in the field highlights the need for developing BP formulations (including adjuvants and carriers) to improve their performance under diverse conditions. For example, innovative materials, like nanocarriers, could help deliver BP more strategically and prolong their bioactivity [102]. Furthermore, cross-regional field trials, particularly within low-income and resource-limited regions, are essential to evaluate the efficacy of these formulations and to characterize the impacts of environmental heterogeneity on their performance.

The estimated BP market accounts for nearly 10% of the global pesticide market [97], and current regulatory evaluations of BP are mainly based on approaches developed for CP [103], which are often not well-adapted. For instance, protocols to assess the hazards of chemical pesticides have limited applicability for living microorganisms [43]. There is thus a need for a more flexible, accessible, and pragmatic framework that can reduce regulatory barriers to the adoption of BP while maintaining appropriate safety standards.

A more holistic risk assessment framework including longer-term impacts would also support the development and more robust evaluation of alternatives, such as BP. New Approach Methodologies (NAMs), including omics, quantitative structure–activity relationships and *in silico* methods, can help reveal ecosystem function alterations within the complex environmental interaction network, which could contribute to predicting and mitigating the risk of potential resistance cycles associated with long-term application of pesticides. More importantly, these non-standard tests could reduce reliance on animal experiments.

Integrating feedback from diverse stakeholders (regulatory authorities, farmers, consumers and scientists), with short-term and long-term evaluations of spatial heterogeneity, economic viability and environmental sustainability, is essential to appropriately balance precautionary principles with the imperative to foster innovation in addressing agricultural and environmental challenges. Agrobiodiversity practices, such as crop diversification (e.g., intercropping) and co-cultures of crops with domestic animals (e.g., rice-fish co-culture), can enhance ecological pest suppression and reduce reliance on pesticide application [104,105]. Within this broader transdisciplinary framework, BP should be strategically integrated into agrobiodiversity-based production systems as a component of sustainable pest management, ultimately enhancing agricultural productivity while promoting long-term environmental sustainability.

## CRediT authorship contribution statement

**Weitao Wang:** Writing – original draft, Visualization, Validation, Software, Methodology, Funding acquisition, Formal analysis, Data curation, Conceptualization. **Sarah J. Knight:** Writing – review & editing, Visualization, Project administration, Data curation. **Yannan Zhou:** Visualization, Validation, Formal analysis, Data curation. **Karin Müller:** Writing – review & editing, Visualization, Validation, Supervision. **Jinshu Chi:** Writing – review & editing, Validation. **Melanie Kah:** Writing – review & editing, Visualization, Supervision, Project administration, Methodology, Conceptualization.

## Declaration of competing interest

The authors declare that they have no known competing financial interests or personal relationships that could have appeared to influence the work reported in this paper.
