# Supplementary material for: Biopesticides vs conventional synthetic pesticides: A multidimensional evaluation of impacts on agricultural productivity and environmental health
Source: Eco Environ Health. 2026 Sep 15;5(4):100286. doi: 10.1016/j.eehl.2026.100286 (PMC13635564; doi:10.1016/j.eehl.2026.100286)
Supplement: Supplementary file 1 [file mmc1.docx]

**SUPPLEMENTARY MATERIALS**

**Biopesticides vs Conventional Synthetic Pesticides: A Multidimensional Evaluation of Impacts on Agricultural Productivity and Environmental Health**

Weitao Wang^a,*^, Sarah J. Knight^b^, Yannan Zhou^c^, Karin Müller^d^, Jinshu Chi^e^, Melanie Kah^a,*^

^a^ The School of Environment, The University of Auckland, Auckland 1010, New Zealand

^b^ The School of Biological Sciences, The University of Auckland, Auckland 1010, New Zealand

^c^ Thrust of Smart Manufacturing, The Hong Kong University of Science and Technology (Guangzhou), Guangzhou 511453, China

^d^ New Zealand Institute for Bioeconomy Science Limited, Plant & Food Research, Auckland 1025, New Zealand

^e^ Earth, Ocean and Atmospheric Sciences Thrust, The Hong Kong University of Science and Technology (Guangzhou), Guangzhou 511453, China

^*^ Corresponding authors: Weitao Wang, Email: [weitao.wang@auckland.ac.nz](mailto:weitao.wang@auckland.ac.nz); Melanie Kah, Email: [melanie.kah@auckland.ac.nz](mailto:melanie.kah@auckland.ac.nz)

**Table of Contents**

**Figure S1.** Overview of screened literature.	S3

**Figure S2.** Improvement difference of Shannon diversity index in bacteria and fungi between biopesticides and conventional pesticides.	S5

**Table S1.** Calculation process of all parameters.	S6

**Table S2.** Values shown in the box plots presented in Figure 2.	S7

**Table S3.** Values shown in the box plots presented in Figure 3.	S8

**Table S4.** Values shown in the box plots presented in Figure 4.	S9

**Table S5.** Revenue of crops treated with commercial pesticide products.	S10

**Table S6.** Values shown in the box plots presented in Figure 6.	S11

**Figure S1.** Overview of the screened literatures. **(a)** Distribution of the screened literature. The horizontal bars on the left indicate the total number of papers that assessed each endpoint, while the vertical bars represent the number of papers addressing unique or combined sets of endpoints, as indicated by the connected dots below. **(b)** Overview of experimental locations. The plots were categorized by climate types based on the Köppen climate classification and World Bank country classifications by income level for 2024-2025. **(c)** The types of crops investigated in the literatures classified according to FAO Classification of Crops.

**Figure S2.** Improvement difference of Shannon diversity index in bacteria (**a**) and fungi (**b**) between biopesticides (BP) and conventional synthetic pesticides (CP). Individual points represent individual observations, open circles indicate the mean values, and error bars indicate the standard error. “ns” indicates that no statistically significant difference was observed between the two treatments.

Table S1. Calculation process of all parameters.

| **Formula** | **Description** |
| --- | --- |
| Efficacy (%) = M_Treatment_ – M_CK_ | M_Treatment_ indicates the mortality of target organisms (%) treated by biopesticides or conventional pesticides, while M_CK_ indicates the mortality in corresponding control groups that received no treatment. |
| Yield improvement (%) = (Y_Treatment_ – Y_CK_) / Y_CK_ | Y_Treatment_ indicates the yield (kg/acre) treated by biopesticides or conventional pesticides, while M_CK_ indicates the yield (kg/acre) in corresponding control groups that received no treatment. |
| Ratio = V_BP_ / V_CP_ | V_BP_ and V_CP_ indicate the values derived from biopesticide and conventional pesticide treatments, respectively (e.g., efficacy, yield improvement) |
| Revenue difference = U × B × \|Y_BP_ –Y_CP_ \| | U indicates the unit prize of the crop, and B indicates the base yield of the crop. Y_BP_ and Y_CP_ represent the yield improvement of BP and CP, respectively. |
| Mortality of Natural Enemies (%) = (N_Treatment_ – N_CK_) / N_CK_ | N_Treatment_ indicates the number of natural enemies treated by biopesticides or conventional pesticides, while N_CK_ indicates the number of natural enemies in corresponding control groups that received no treatment. |
| Diversity improvement (%) = (S_Treatment_ – S_CK_) / S_CK_ | S_Treatment_ indicates the Shannon diversity index treated by biopesticides or conventional pesticides, while S_CK_ indicates the Shannon diversity index in corresponding control groups that received no treatment. |
| Soil component improvement (%) = (C_Treatment_ – C_CK_) / C_CK_ | C_Treatment_ indicates the soil component content treated by biopesticides or conventional pesticides, while C_CK_ indicates the soil component content in corresponding control groups that received no treatment. |

Table S2. Values shown in the box plots presented in Figure 2.

|  | Total | | Fungicide | | Insecticide | | Herbicide | |
| --- | --- | --- | --- | --- | --- | --- | --- | --- |
|  | Biopesticide | Pesticide | Biopesticide | Pesticide | Biopesticide | Pesticide | Biopesticide | Pesticide |
| Number of values | 608 | 608 | 327 | 327 | 252 | 252 | 29 | 29 |
| Minimum | 0 | 0 | 0 | 0 | 0 | 0 | 0 | 22.00 |
| 25% Percentile | 23.49 | 31.06 | 28.47 | 31.81 | 12.80 | 22.00 | 33.23 | 56.49 |
| Median | 48.02 | 60.99 | 44.31 | 55.03 | 54.40 | 62.62 | 62.44 | 91.30 |
| 75% Percentile | 71.40 | 83.55 | 68.92 | 71.33 | 74.53 | 91.90 | 89.04 | 96.92 |
| Maximum | 100.0 | 100.0 | 100.0 | 100.0 | 100.0 | 100.0 | 97.56 | 98.17 |
| Mean | 48.43 | 56.49 | 47.72 | 53.03 | 47.74 | 58.28 | 59.35 | 77.76 |
| Std. Deviation | 28.88 | 29.70 | 24.75 | 25.69 | 32.87 | 33.71 | 32.77 | 24.97 |

Table S3. Values shown in the box plots presented in Figure 3.

|  | **Laboratory** | | **Semi-field** | | **Field** | |
| --- | --- | --- | --- | --- | --- | --- |
|  | Biopesticide | Pesticide | Biopesticide | Pesticide | Biopesticide | Pesticide |
| Number of values | 31 | 31 | 181 | 181 | 396 | 396 |
| Minimum | 4.160 | 0 | 0 | 0 | 0 | 0 |
| 25% Percentile | 52.10 | 43.67 | 17.44 | 16.70 | 25.71 | 34.82 |
| Median | 78.10 | 78.50 | 31.80 | 42.42 | 50.01 | 62.62 |
| 75% Percentile | 96.60 | 91.00 | 72.20 | 81.93 | 69.65 | 83.19 |
| Maximum | 100.0 | 100.0 | 99.78 | 100.0 | 100.0 | 100.0 |
| Mean | 69.75 | 67.23 | 44.38 | 51.05 | 48.60 | 58.13 |
| Std. Deviation | 30.25 | 29.51 | 31.04 | 31.84 | 27.01 | 28.32 |

Table S4. Values shown in the box plots presented in Figure 4.

|  | **Total** | | **Fungicide** | | **Insecticide** | | **Herbicide** | |
| --- | --- | --- | --- | --- | --- | --- | --- | --- |
|  | Biopesticide | Pesticide | Biopesticide | Pesticide | Biopesticide | Pesticide | Biopesticide | Pesticide |
| Number of values | 353 | 353 | 206 | 206 | 139 | 139 | 8 | 8 |
| Minimum | 0 | 0 | 0 | 0 | 0 | 0 | 0.980 | 0 |
| 25% Percentile | 2.530 | 3.760 | 5.165 | 6.408 | 0.000 | 0.000 | 8.490 | 6.180 |
| Median | 10.30 | 10.62 | 11.22 | 13.88 | 5.770 | 5.700 | 29.39 | 36.42 |
| 75% Percentile | 23.64 | 23.51 | 23.64 | 23.51 | 22.01 | 27.50 | 72.10 | 83.10 |
| Maximum | 107.0 | 104.9 | 107.0 | 104.8 | 94.98 | 104.9 | 100.6 | 98.24 |
| Mean | 16.51 | 19.23 | 16.79 | 19.45 | 14.78 | 17.47 | 39.37 | 44.12 |
| Std. Deviation | 19.57 | 23.53 | 18.48 | 21.22 | 19.27 | 25.05 | 35.65 | 38.87 |

Table S5. Revenue of crops treated with commercial pesticide products.

| **Crop** | **Advantage Group** | **Unit Prize (USD/kg)** | **Average base yield (ton/ha)** | **Revenue difference (10^3^ USD/ha)** |
| --- | --- | --- | --- | --- |
| Pumpkin | BP | 1.91 | 20.78 | 49.47 |
| Watermelon | BP | 1.46 | 14.15 | 27.49 |
| Papaya | BP | 2.47 | 52.80 | 78.40 |
| Sorghum | BP | 1.47 | 2.98 | 0.65 |
| Rice | BP | 1.33 | 4.27 | 2.29 |
| Potato | BP | 2.94 | 23.14 | 50.82 |
| Alfalfa | BP | 11.48 | 11.42 | 108.92 |
| Cotton | BP | 5.43 | 2.44 | 14.22 |
| Tomato | CP | 4.28 | 4.77 | 36.71 |
| Bean | CP | 3.38 | 1.60 | 14.28 |
| Canola | CP | 1.37 | 1.01 | 3.68 |
| Mung bean | CP | 3.37 | 1.20 | 0.62 |
| Blueberry | CP | 7.40 | 5.09 | 75.18 |
| Strawberry | CP | 5.88 | 45.72 | 176.41 |
| Barley | CP | 1.45 | 5.85 | 4.95 |
| Maize | CP | 0.63 | 4.17 | 0.26 |
| Oats | CP | 1.94 | 6.92 | 9.61 |
| Triticale | CP | 1.92 | 5.80 | 6.89 |
| Wheat | CP | 1.44 | 5.37 | 15.03 |

Table S6. Values shown in the box plots presented in Figure 6.

|  | **Non-targeted Animals** | | **Microbial Diversity** | | **Soil Fertility** | |
| --- | --- | --- | --- | --- | --- | --- |
|  | Biopesticide | Pesticide | Biopesticide | Pesticide | Biopesticide | Pesticide |
| Number of values | 160 | 160 | 27 | 27 | 20 | 20 |
| Minimum | 0 | 0 | -43.01 | -59.06 | -34.40 | -80.57 |
| 25% Percentile | 5.085 | 33.99 | -10.30 | -6.944 | -0.08500 | -9.600 |
| Median | 16.15 | 46.28 | 1.113 | -3.195 | 12.45 | 12.25 |
| 75% Percentile | 34.47 | 82.02 | 3.750 | 2.633 | 63.33 | 25.00 |
| Maximum | 99.39 | 100.0 | 49.14 | 37.46 | 260.0 | 41.70 |
| Mean | 23.50 | 56.17 | -1.377 | -2.838 | 43.36 | 5.373 |
| Std. Deviation | 23.60 | 25.88 | 16.75 | 17.80 | 72.84 | 30.63 |
